# Antiproliferative and Immunoregulatory Effects of Azelaic Acid Against Acute Myeloid Leukemia *via* the Activation of Notch Signaling Pathway

**DOI:** 10.3389/fphar.2019.01396

**Published:** 2019-11-29

**Authors:** Zhang Dongdong, Yanxia Jin, Tian Yang, Qian Yang, Balu Wu, Yanling Chen, Ziyi Luo, Li Liang, Yunjiao Liu, Anjie Xu, Xiqin Tong, Can Can, Lu Ding, Honglei Tu, Yuxin Tan, Hongqiang Jiang, Xiaoyan Liu, Hui Shen, Li Liu, Yunbao Pan, Yongchang Wei, Fuling Zhou

**Affiliations:** ^1^Department of Hematology, Zhongnan Hospital, Wuhan University, Wuhan, China; ^2^Key Laboratory of Artificial Micro- and Naso-Structures of Ministry of Education, School of Physics and Technology, Wuhan University, Wuhan, China; ^3^State Key Laboratory of Virology, College of Chemistry and Molecular Sciences, Wuhan University, Wuhan, China; ^4^Department of Laboratory Medicine, Zhongnan Hospital, Wuhan University, Wuhan, China; ^5^Department of Radiation and Medical Oncology, Zhongnan Hospital, Wuhan University, Wuhan, China

**Keywords:** azelaic acid, acute myeloid leukemia, immunoregulatory, Notch signaling pathway, Notch agonist

## Abstract

Acute myeloid leukemia (AML) is a common type of hematological malignancy that can progress rapidly. AML has a poor prognosis and a high incidence of relapse due to therapeutic resistance. Azelaic acid (AZA), a small molecular compound is known to exhibit antitumor effect on various tumor cells. This study aimed to evaluate the antiproliferative and immunoregulatory effects of AZA against AML*via*the activation of the notch signaling pathway. We found that AZA can inhibit the proliferation of AML cells. In addition, laser confocal microscopy showed AZA-treated AML cells began to swelling and undergo cytoplasmic vacuolization. Importantly, AZA promoted the proliferation of NK and T cells and increased the secretion of TNF-αand IFN-γ. AZA also increased the expression levels of CD107a and TRAIL in NK cells, and CD25 and CD69 in T cells to influence their activation and cytotoxic ability. AZA-treated NK cells can kill AML cells more efficiently at the single-cell level as observed under the microfluidic chips. Further mechanistic analysis using protein mass spectrometry analysis and Notch signaling reporter assay demonstrated that Notch1and Notch2 were up-regulated and the Notch signaling pathway was activated. Moreover, combining AZA with the Notch inhibitor, RO4929097, decreased the expression of Notch1and Notch2, and downstream HES1 and HEY1, which rendered AML cells insensitive to AZA-induced apoptosis and alleviated AZA-mediated cytotoxicity in AML. *In vivo*, AZA relieved the leukemic spleen infiltration and extended the survival. The percentage of CD3^-^CD56^+^NK cells and CD4^+^CD8^+^T cells as well as the secretion of cytotoxic cytokines was increased after the treatment of AZA. The overall findings reveal that AZA is a potential Notch agonist against AML in activating the Notch signaling pathway.

## Introduction

Acute myeloid leukemia (AML) is a hematological malignancy arising from hematopoietic stem cells. AML is a common form of acute leukemia in adults with poor prognosis. Only 25% of patients survival 5 years after their diagnosis (Beyar-Katz and Gill, 2018) and AML treatment remains largely unchanged over the past several decades. High dose chemotherapy for inhibiting the accumulation of leukemic blasts, consolidation chemotherapy, and a stem cell transplant during remission remain as the main methods of AML treatment. However, the remission method is difficult to maintain without subsequent treatment and the toxic side effects of chemotherapy, such as the myelosuppression or subsequent severe infection, render some patients intolerable to treatment. Although some novel targeted therapies and chemotherapeutic agents benefit more AML patients, overall survival remains low due to drug resistance and disease recurrence (Yanada and Naoe, 2012).

Notch signaling is a conserved cell-to-cell pathway that participated in different functions, such as stem cell maintenance, cell fate, apoptosis, and the regulation of immunity. The aberrant activation of the Notch signaling pathway has contributed to many solid tumors and T cell acute lymphoblastic leukemia (Espinoza and Miele, 2013), but Notch receptor activation and the expression of Notch downstream targets were found to be low in AML samples. Importantly, the reactivation of Notch can inhibit the proliferation of AML cells and can lead to the loss of B cell lymphoma 2 (Bcl-2) and the upregulation of p53, thereby inducing the caspase-dependent apoptosis (Kannan et al., 2013; Lobry et al., 2013). Additionally, the intracellular Notch 1 (ICN1) downstream target, HES-1, can bind to the promoter region of the*FLT3*gene, the activation of Notch can exert an antileukemic effect by repressing the expression of*FLT3* (Kato et al., 2015).

The Notch signaling pathway plays a substantial role in regulating the development and functions of immune cells (Radtke et al., 2013). Notch1 signaling is thus involved in the generation and differentiation of CTL (Cho et al., 2009; Kuijk et al., 2013), while Notch2 signaling played a crucial role in CTL cytotoxic response by promoting the differentiation of CTL and directly regulating granzyme B and perforin expression (Maekawa et al., 2008; Sugimoto et al., 2010). Additionally, Notch2 signaling is concerned involved the development and maturation of human NK cells (Felices et al., 2014; Kyoizumi et al., 2017). Prior studies have shown that Jagged2–Notch can enhance the antitumor cytolytic activity of NK *in vitro* and *in vivo* (Kijima et al., 2008). AML cells are susceptible to T cell recognition and attack as they express major histocompatibility complex (MHC) classes I and II. AML cells are also susceptible to NK cell attack as they express MIC-A/B to activate the NK receptor, NKG2D (Barrett and Le Blanc, 2010); hence, the activation of Notch can enhance the cytotoxicity of NK and T cells to AML. As such, we believe that targeting Notch not only inhibits the proliferation of AML cells, but also improves the immunologic function which can benefit more AML patients.

AZA is a nine-carbon dicarboxylic acid that has antimicrobial and anti-inflammatory properties and is used to treat some skin diseases, such as acne and rosacea (McGee and Wilkin, 2018). AZA exerts antitumor effect on several tumor cells, such as human melanoma (Fitton and Goa, 1991) and human T lymphotropic virus I (HLTV-1) infected T-cell leukemia (U-Taniguchi et al., 1995). Early studies have shown that AZA can scavenge reactive oxygen species (ROS) and decrease the superoxide anion and other free radicals (Akamatsu et al., 1991; Passi et al., 1991). Excessive H_2_O_2_ triggered the up-regulation of oncogene c-Jun activation domain-bind protein-1 (*Jab1*) and thioredoxin-1 (*Trx1*) which are related to the poor prognosis of AML (Pan et al., 2017). AZA had a dose- and time-dependent cytotoxic effects on AML cell lines and can sensitize AML cells to chemotherapy; more importantly, AZA can inhibit the expression of Trx/Jab1 against AML cells (Pan et al., 2017). However, the molecular basis for the antileukemic effects of AZA is unknown. In the present study, we investigated the antileukemic activity and immunoregulatory effect of AZA and explored its potential mechanism.

## Materials and Methods

### Materials

AZA (Cat# 95054) and DMSO (Cat# D2650) were from Sigma (USA). Notch inhibitor RO4929097 was from Selleck (Cat# 847925-91-1). The following antibodies were used: CD3(Cat# 100203), CD4 (Cat# 100431), CD8 (Cat# 100761), CD107a (Cat# 328605), TRAIL (Cat# 308205), CD25 (Cat# 302605), and CD69 (Cat# 310903) were purchased from BioLegend (USA), ICN1 (Cat# CSB-PA084572), ICN2 (Cat# CSB-PA964902) were from CUSABIO (USA); GAPDH (Cat# 6004-1), β-actin (Cat# 14395-1) were from ProteinTech (USA). TNF-α (Human Cat# 1117202, Mouse Cat# 1217202) and IFN-γ (Human Cat# 1110002, Mouse Cat# 1210002) enzyme-linked immunosorbent assay kits were purchased from DAKEWEI (China). Human Notch Pathway Reporter kit was from BPS Bioscience (Cat# 79503).

### Cell Culture and Cell Isolation

The AML cell lines, HL60, THP-1, U937, Molm-13, NB4, and Human AML cells were maintained in laboratory. All the cells were grown in RPMI-1640 medium with 10% fetal bovine serum (FBS) (Gibco, Grand Island, NY) and 1% penicillin streptomycin solution. The C1498 cell lines and 293T cells were purchased from ATCC and were cultured in DMEM. The NKL cell line was a gift from Bei Du Biotech (Wuhan, PR China). It was maintained in the laboratory and cultured in complete IL-2-containing (100 IU/mL) RPMI-1640 medium.

Peripheral blood was obtained from 10 healthy adult donors who provided written informed consent for participation in this study. Peripheral mononuclear cells (PBMC) were isolated by Ficoll-Hypaque gradient centrifugation (TBD sciences, Cat# HY2015, China). Furthermore, human NK cells were enriched from PBMC with NK-cell Enrichment Cocktail (StemCell, Cat#15065, Canada) and the NK cells sorted as CD45^+^CD3^-^CD56^+^on fluorescence-activated cell sorter.

The freshly isolated PBMCs were cultured in RPMI-1640 medium supplemented with 10% PBS, IL-2 (100 IU/ml), 0.1 mM of nonessential amino acid solution, 50 µM of 2-mercaptoehanol, and CD3/CD28 T cell activator (Stemcell, Cat# 10971, Canada) following the exchange of the cell medium every 3 days, twice. After dendritic cells (DCs) grew against the wall of the flask, the resting suspension cells obtained was T cells. AML patient primary cells (AML-PC) were isolated from the AML patients’ bone marrow samples by lymphocyte isolation. Importantly, all subjects gave written informed consent in accordance with the recommendations of the Ethics and Scientific Committee of Zhongnan Hospital of Wuhan University and the Declaration of Helsinki.

### Cell Proliferation

AML cells, NK, or T cells (1.0×10^4^/well) were seeded in 96-well plates at 37 °C in a 5% CO_2_ incubator for 24 h. The cells were counted after trypan blue staining. Each sampling was performed in triplicate and each well was counted three times. The cell proliferation assays were performed using a Cell Counting Kit-8 (Dojindo, Cat# JE603, Japan). AZA at different concentration was added and cultured 2 days. Thereafter, 10 µl CCK8 was added and the plates were incubated for additional 4 h. The absorbance reading at 450 nm in each well was determined with a microplate reader (SpectraMax M2, Molecular Devices, China).

### Synthesis of AZA With Fluorophores and Fluorescence Confocal Microscopy

To observe the changes in cell morphology after AZA treatment, a fluorophore was attached to AZA. We found that 4,4-difluoro-8-(4-hydroxy) phenyl-1,3,5,7-tetramethyl-4-bora-3a,4a-diaza-s-indacene (BDP-OH) can combine perfectly with AZA without affecting the physicochemical properties of the drug. In addition, we synthesized BDP-AZA which can emit a green light under the excitation of blue light. The detailed steps for the synthesis are presented in **Additional File 1**.

Apoptotic cell morphology was detected by a confocal microscope. HL60 cells and THP-1 cells were seeded in 10 mm laser confocal dish (Corning, USA) with 1mL RPMI-1640 over night. BDP-AZA (1 mM) was then added before dynamic observation. The dynamic changes in the cell morphology at different time points were observed with a confocal microscope (Nikon, AIR) and the images were captured using NIS-Elements (AR 4.50.00).

### Cytotoxicity Assay

Cytotoxicity was determined with an LDH Cytotoxicity assay Kit (Beyotime, Cat# C0017, China). The effector cells were treated with AZA for 48 h before the start of the experiment. C1498, U937, and NB4 cells were used as the target cells and placed in 96-well plates at 4.0×10^3^/well. Effector cells were added to target cells at effector/target (E:T) ratio of 10:1 and 5:1, respectively. The effector and target cell mixtures were incubated for 6 h. Thereafter, the supernatant was separated after centrifugation and moved to a new plate, LDH working reagent (60 µl) was added and the plates were incubated for additional 1 h. The absorbance (A) at 490 nm in each well was determined with a microplate reader. Cytotoxicity (%) = [(A of target plus effector cells − A of effector cells)/A of target cells] × 100%.

### Co-Culture Assay

Cell viability was determined by using the Alamar Blue assay (Yeasen, Cat# 40202ES60, China). NK cells or T cells were placed under treatment with AZA or PBS for 48 h and the supernatant was harvested for the following experiment. The THP-1, U937, and Human AML cells were then co-cultured with the collected supernatant in 96-well plates for 48 h. Subsequently, 10 µl Alamar Blue was added to each well and the plates were incubated to record measurements at different time points. The absorbance at 560 nm in each well was determined with a microplate reader.

### CM-Dil Living Cell Staining and Microfluidic Single-Cell Technology

Chlormethylbenzamido-1,1-dioctadecyl-3,3,3',3'-tetramethylin-docarbocyamine (CM-Dil) is a type of tracer that is commonly used for labeling cellular membrane. Cells were centrifuged at 1,000 rpm for 5 min at 4°C and washed with PBS twice, and then incubated with 5 µl CM-Dil dye (ThermoFisher, Cat# C7001, USA) at 37°C for 5 min and 4°C for 15 min. Under the excitation wavelength, the cell membranes displayed orange-red color when excited by green light.

To detect the cytotoxicity of NK cells after AZA treatment at the single-cell level, the microfluidics chips were designed for single-cell capture as previously described (Liang et al., 2018; Yan et al., 2018). AML cells with CM-Dil staining were injected into the chip and fixed in one inlet. After AZA treatment, NK cells were injected from another inlet and the AML cells and NK cells were fixed in the middle of the chips and contacted with each other. The dynamic changes in cell morphology at different time points were observed by microscopy (NIKON, ECLIPSE TiU) and the images were acquired with a sCMOS camera (Hamamatsu, ORCA-Flash 4.0 v2).

### Cytokine ELISA

NK cells or T cells (1.0×10^6^/well) were treated with AZA or Notch inhibitor, RO4929097, for 48 h following by plating in the 6-well plates with THP-1 cells at an E:T ratios of 3:1. After 24 h of co-culture, TNF-α and IFN-γ levels in cell culture supernatants were evaluated by commercial enzyme-linked immunosorbent assay (ELISA) kits according to the manufacturer's procedure.

### Protein Mass Spectrometry Analysis

Proteins of the AZA-treated group and the blank group were degraded to peptides by tryptic digestion, and the peptides from two different samples were labeled with isotopomeric dimethyl labels and analyzed using a hybrid Quadrupole-TOF LC-MS/MS Mass Spectrometer (TripleTOF 5600, AB Sciex Instruments). Raw mass spectrometry data were submitted to BGI WEGO2.0 (http://biodb.swu.edu.cn/cgi-bin/wego/index.pl) to analyze the function of the differential proteins.

### Notch Reporter Assay

Notch activity was measured on 293T cells using Human Notch Pathway Reporter kit, according to the manufacturer's recommendation. Briefly, 1.0×10^5^ cells/well were transfected with the Notch reporter or controls, and then treated with DMSO or 10 µM RO4929097 or 10 µM AZA for 24 h. The Notch pathway activity was measured by Luciferase reporter gene assays.

### Western Blot Analysis

Protein was extracted by RIPA buffer and PMSF (BD Biosciences, USA) derived from AML cells in the log phase of growth. Total protein (20 µg) was resolved by SDS gel electrophoresis and transferred to nitrocellulose membranes. Membranes were incubated with the following primary antibodies at 4°C overnight: ICN, ICN2, and β–actin antibodies (1:5000). Additionally, the membranes were incubated with HRP-conjugated goat antirabbit IgG (diluted 1:5000, ProteinTech) and goat antimouse IgG (diluted 1:5000, ProteinTech) secondary antibodies for 60 min at 25°C and detected with the Immobilon™ Western Chemiluminescent HRP Substrate (Millipore, Billerica, USA) using a Western Chemiluminescent Imaging System (Tanon 5200).

### Flow Cytometry Analysis

NKL cells or T cells were treated with AZA for 48 h then harvested. NK and T cells in mice were collected from the splenic mononuclear cells (MNC). To detect the expression levels of cytotoxicity-associated molecules, NKL cells were stained with FITC-conjugated CD107a or PE-conjugated TRAIL, and T cells were stained with FITC-conjugated CD69 or PE-conjugated CD25. The NK cells in mice were stained with PE-conjugated CD56, while, T cells were stained with FITC-conjugated CD3, PERCP-conjugated-CD4, and PE-conjugated CD8 at 37°C for 30 min. Stained cells were analyzed using a flow cytometer, and data were analyzed using CytExpert 2.0 software and Flowjo 10 software.

### RNA Isolation, Cdna Preparation, and Quantitative PCR

AML cells were seeded and allowed to grow for 24 h, and then treated with AZA for 48 h. Cells were homogenized in Trizol (BD Biosciences, USA) and total RNAs were isolated according to the manufacturer’s recommendations. In addition, cDNAs were synthesized using the PrimeScript™ RT reagent kit with gDNA Eraser (Takara, Japan) and amplified with the SYBR GREEN MIXTURE kit (Bio-Rad, USA) in a final volume of 20 µl containing 1 µl each primer, 12.5 µl mixture, and 8.5 µl ddH_2_O. Thirty-five to thirty-nine amplification cycles were performed at 95°C for 10 min and 15 sec; and 60°C for 15 sec. Primers are listed in **Additional File 2**.

### Animal Studies

All animal experiments were approved by the Animal Care and Use Committee of Wuhan University. C1498 cells (4×10^6^per mouse) were injected into C57BL/6 mice (female, 4–5 weeks) through the tail-vein for the development of leukemic disease, and then randomly assigned to two experimental groups(i.e., saline and AZA) with six mice in each group. When WBC counting and peripheral blood cell smear test indicated the development of leukemia, 10 mg/kg AZA/per mouse was intraperitoneally injected once every 3 days. However, for the survival studies, mice were sacrificed when any signs of distress were recognized (i.e., immobility and extreme emaciation). Importantly, all animal studies were performed according to the Institutional Animal Care and Use Committee of Wuhan University (2017048).

### Wright’s Staining, Hematoxylin, and Eosin (H&E) and Immunohistochemistry

The collected blood and bone marrow from the animal studies were used to create blood smear and bone marrow smear by Wright's staining. Tissues collected from mice were cut, dewaxed, and hydrated. Some samples were also stained with H&E while some samples were under pretreatment for antigen retrieval within citrate buffer at pH 6.0 at 100°C for 30 min. The primary antibodies (Notch1 antibodies, 1:150; Notch2 antibodies,1:100) were then blocked overnight at 4°C, followed by HRP-conjugated secondary antibody. All slides were viewed and photographed with a Nikon microscope mounted with a high-resolution spot camera.

### Statistical Analysis

The sample sizes for each study were sufficiently chosen to allow statistical analysis of the outcomes of the experimental versus control of the studies based on literature documentation of similar well-characterized experiments. Additionally, *in vitro* experiments, such as qPCR and CCK-8 assay were routinely repeated at least three times unless indicated in figure legends or main text. The statistical analysis was performed using Student’s t-test and analysis of variance (ANOVA). All analyses were performed using the GraphPad Prism 5 Software. P < 0.05 indicated statistical significant and the survival time of mice was analyzed by the Kaplan-Meier method.

## Results

### Aza Inhibits Aml Cell Viability

**Figure 1:**
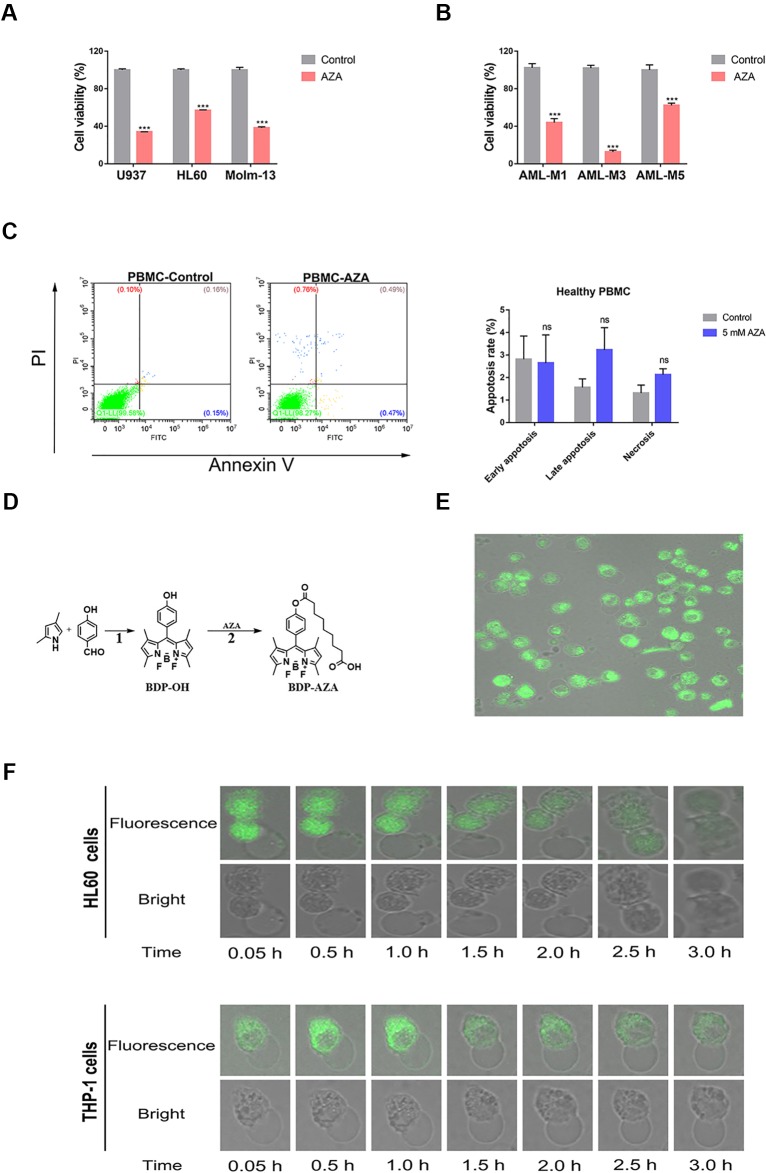
Azelaic acid (AZA) inhibits acute myeloid leukemia (AML) cell proliferation. **(A)** The U937, HL60, and Molm-13 cells were treated with AZA at concentration of 5.0 mM for 48 h. Cell viability was measured by the CCK-8 method. **(B)** AML patient cells were isolated and then treated with 5 mM AZA for 48 h. Cell viability was measured by the CCK-8 method. **(C)** Peripheral mononuclear cells (PBMC) were treated with 5 mM AZA and then stained with Annexin V/PI. The apoptotic rate was measured by flow cytometry analysis. **(D)** The synthetic method of BDP-AZA. **(E)** HL60 cells were cultured in confocal dish for 24 h and 1 mM BDP-AZA was added to the medium. Thereafter, cell membrane was observed to turn green with confocal microscope under the excitation of blue light. **(F)** HL60 and THP-1 cells began to develop swelling and cytoplasmic vacuolization under fluorescence confocal microscopy after AZA treatment. A total of three independent experiments were performed, ***P < 0.001. ns, no significance.

A previous study demonstrated that AZA can inhibit the proliferation of AML cells at low micromolar level (Pan et al., 2017) and our experimental results further verified this conclusion. Notably, AZA displayed cytolytic activity on all tested AML cell lines and AML patient cells. Cell viability after treatment with 5 mM AZA was nearly 34% (U937), 57% (HL60), 37% (Molm-13), 44% (AML-M1), 12% (AML-M3), and 65% (AML-M5), respectively (**Figures 1A, B**). However, we did not observe any obvious apoptosis in healthy PBMC at the same AZA concentration (**Figure 1C**), suggesting that AZA can selectively inhibit the proliferation of AML cells. Furthermore, to clarify the observed cell morphology after the entry of drugs into the cell, AZA was subjected to a fluorescent modification, without alteration to its structure and function (**Figure 1D**). The fluorescent modified BDP-AZA appeared green under the excitation of blue fluorescence (**Additional File 1: Figure S1**). In addition, we found when BDP-AZA was added to the medium, cell membrane instantly turned green and gradually spread to the whole cell (**Figure 1E**). Subsequently, the green fluorescence gradually faded and disappeared completely at 3 h. Interestingly, cells began to develop swelling and cytoplasmic vacuolization as revealed by the fluorescence confocal microscopy (**Figure 1F**).

### AZA Promotes the Proliferation of Immunologic Effector Cells and Enhances the Generation of the Cytolysis-Associated Cytokines

**Figure 2:**
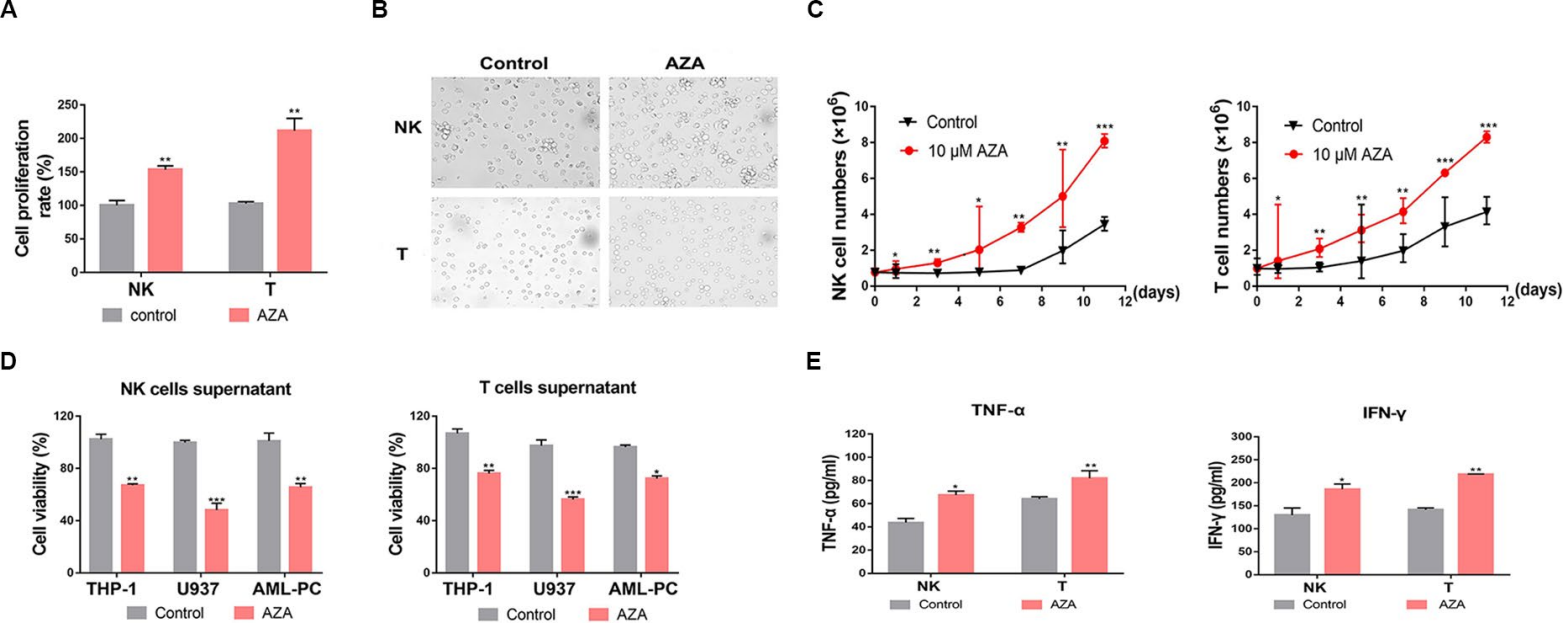
Azelaic acid (AZA) promotes cell proliferation of NK and T cells and increases the secretion of cytokines. **(A)** NK and T cells were treated with 10 µM AZA for 24 h and cell proliferation rates were measured by the CCK-8 methods. **(B)** NK and T cells were treated with DMSO and 10 µM AZA for 48 h and then observed under a microscope. **(C)** NK and T cells were treated with 10 µM AZA for 12 days. Cell proliferation rates were measured and cumulative growth curve of NK or T cells was derived. **(D)** NK and T cells were pre-treated with 10 µM AZA before the collected supernatants were co-cultured with the AML cell lines and the human acute myeloid leukemia (AML) patient cells. The viability of AML cells was measured by Alamar Blue assay. **(E)** NK and T cells were treated with 10 µM AZA for 48 h before co-culture with THP-1 cells at an E:T ratio of 3:1 for 6 h, the level of TNF-α and IFN-γ in the supernatants was measured by ELISA. Data represent standard deviations for three independent experiments. Compared to “control” group (student’s test), *P < 0.05, **P < 0.01,***P < 0.001, E:T, the ratio of effector cell and target cell; AML-PC, primary AML patient cells.

NK and T cells were pre-treated with AZA at different times and doses before the cell viability was measured by CCK-8 methods. We found that AZA can promote the proliferation of NK and T cells at the optimum concentration of 10 µM (**Figure 2A**). AZA promoted nearly a 1.5 fold increase in NK cells and 2.1 fold increase in T cells in 24 h. In addition, after simulation for 2 days, NK cells and T cells became highly proliferative, where NK cells formed some cell colonies and T cells became enlarged (**Figure 2B**). By maintaining stimulation with AZA consistently for 12 days, cell viability and proliferation were monitored by Trypan Blue exclusion test every 2 days. Notably, we found AZA could promote nearly 2.6-fold higher proliferation rate than the un-stimulated group (**Figure 2C**).

NK and T cells could mediate cytotoxicity depending on the secretion cytolysis-related cytokines. We collected the supernatants of NK and T cells and detected whether the supernatants had an anti-leukemic effect. The supernatants were harvested when the effector cells were treated with AZA for 48 h. The collected supernatant was co-cultured with THP-1, U937, and human AML cells with fresh RPMI-1640 medium for 24 h. The AML cell viability was measured by an Alamar Blue assay. The result showed that the AZA-treated supernatant had a stronger antileukemic effect (**Figure 2D**). Thus, to provide a suitable reasoning, we analyzed the levels of IFN-γ and TNF-α in the supernatant. As shown in **Figure 2E**, the IFN-γ and TNF-α levels produced by AZA-treated NK cells co-cultured with THP-1 cells were 186 ± 19 pg/ml and 64 ± 2 pg/ml, respectively, and those produced by AZA-treated T cells were 218 ± 2 pg/ml and 82 ± 7 pg/ml, respectively, these values, which were significantly higher than those in control group. These results indicate that AZA can promote the proliferation of effector cells and increase the secretion of cytolysis-related cytokines against AML cells.

### AZA Promotes the Activation of NK and T Cells

**Figure 3:**
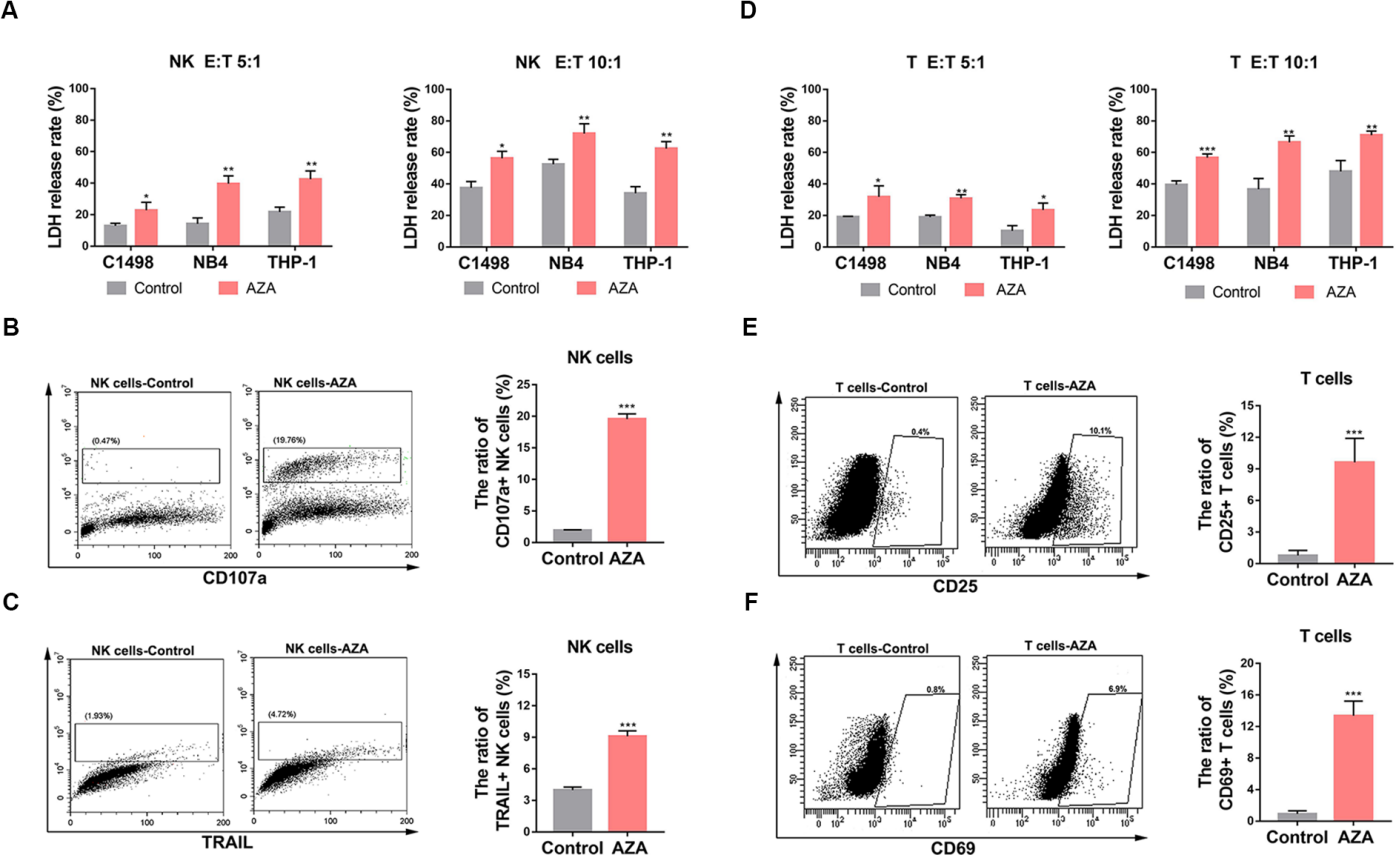
Azelaic acid (AZA) promotes the activation of NK and T cells and enhances cytotoxicity against acute myeloid leukemia (AML) cells. **(A, D)** NK and T cells were treated with 10 µM AZA for 48 h before incubation with AML cell lines at different E:T ratio for the cytotoxicity assay (E:T = 5:1 and 10:1). Cytotoxicity was measured by an LDH assay. **(B**, **C)** NK cells were treated with 10 µM AZA for 48 h before incubation with the AML cell lines at an E:T ratio of 3:1. Cells were then stained with CD107a and TRAIL for flow cytometric analysis. Right, the percentage of positive staining cells. **(E, F)** T cells were treated with 10 µM AZA for 48 h before incubation with the AML cell lines at an E:T ratio of 3:1. Cells were then stained with CD25 and CD69 for flow cytometric analysis. Right, the percentage of positive staining cells. A total of three independent experiments were performed, diagrams represents mean percentage, statistical significance was determined with student's test. *P < 0.05, **P < 0.01, ***P < 0.001.

To evaluate whether AZA treatment increased the susceptibility of AML cells to NK and T cell-mediated cytotoxicity, 10 µM AZA was used to treat NK and T cells for 48 h. Effector cells then were co-cultured with AML cells at different ratios for 6 h. AML cell viability was measured by LDH assay. As shown in **Figure 3**, NK (**Figure 3A**) and T cells (**Figure 3D**) treated with AZA had a significantly higher cytotoxicity than without AZA treatment.

The expression levels of cytolysis-related receptors and molecules are known to influence NK and T cells activation and cytotoxic ability. To investigate the mechanism how AZA improves the cytotoxicity of NK and T cells, we analyzed the general and early activation markers of T cells (CD25 and CD69, respectively) and the general activators of NK cells (TRAIL and CD107a). The results showed that the percentage of CD107a and TRAIL in NK cells (**Figures 3B, C**) and CD25 and CD69 in T cells (**Figures 3E, F**) in the AZA-treated group were higher than those in the control group. This finding indicated that AZA can promote the activation of NK and T cells against AML.

### Validation of AZA Sensitive the Cytotoxicity of NK Cells on the Single-Cell Level

**Figure 4:**
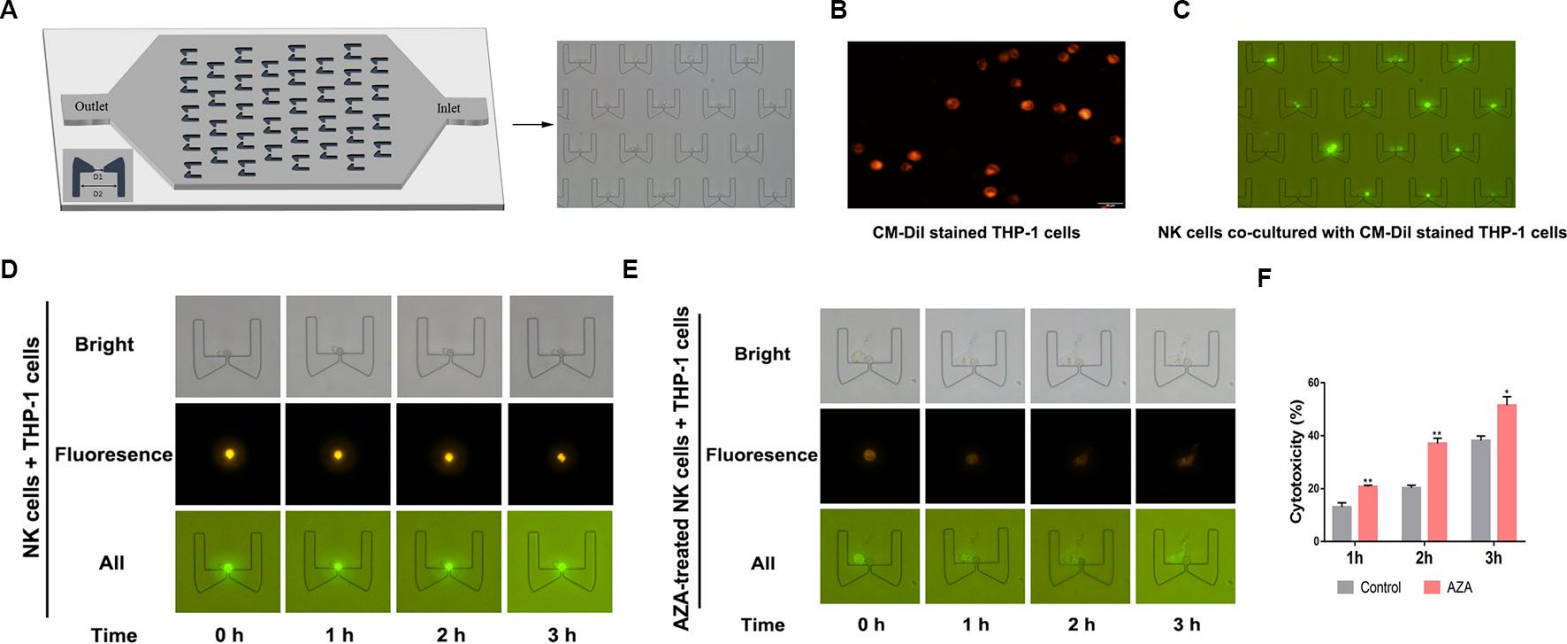
Azelaic acid (AZA) enhanced the cytotoxicity of NK cells against acute myeloid leukemia (AML) cells. **(A)** The micro-fluidic chips used for trapping cells and cytotoxic analysis, D1 = 5 µm and D2 = 50 µm. The NK cells and the target cells can be trapped from two opposite directions between the clips. **(B)** The CM-Dil stained THP-1 cells membrane can display an orange-red color with the excitement of green light. **(C)** The fluorescent THP-1 cells (CM-Dil stained) and NK cells (none stained) were trapped in the micro-fluidic chip and observed with a fluorescence microscope. **(D**, **E)** NK cells and AZA-treated NK cells were in contact with trapped THP-1 cells. The THP-1 cell motion morphology was observed on the micro-fluidics chip with a fluorescence microscope. **(F)** The total number of CM-Dil stained membrane rupture cells in different fields at different times were observed under the microscope. Data represent standard deviations for three independent experiments. Statistical significance was determined with student's test. *P < 0.05, **P < 0.01.

To observe how AZA can sensitize the antileukemic activity of effector cells, we designed a microfluidic chip which can trap cells from two opposite directions. Depending the cell size, the NK cells were trapped from one direction and the target cells were trapped from the opposite direction. The trapped cells can be contacted with each other between the clips (**Figure 4A**). Hence, to distinguish between the NK cells and target cells, to further observe the tumor-killing effect of NK cells, the target cells were prestained with a CM-Dil dye, to distinguish the target cells, which could be observed as the membrane displayed orange-red color with green fluorescence microscope (**Figure 4B**). Notably, the CM-Dil stained THP-1 cells were distinguished from the label-free NK cells based on the fluorescence under the optical filter (**Figure 4C**), and after continuous observation for 3 h, we found that the morphology of THP-1 cells developed swelling and lysis. Total number of CM-Dil stained membrane rupture cells in different fields at different times was observed under the microscope (**Figures 4D, E**) and the statistical results were analyzed (**Figure 4F**). The result showed that AZA-treated NK can kill AML cells faster and more efficient.

### AZA Induces the Up-Regulation of Notch

**Figure 5:**
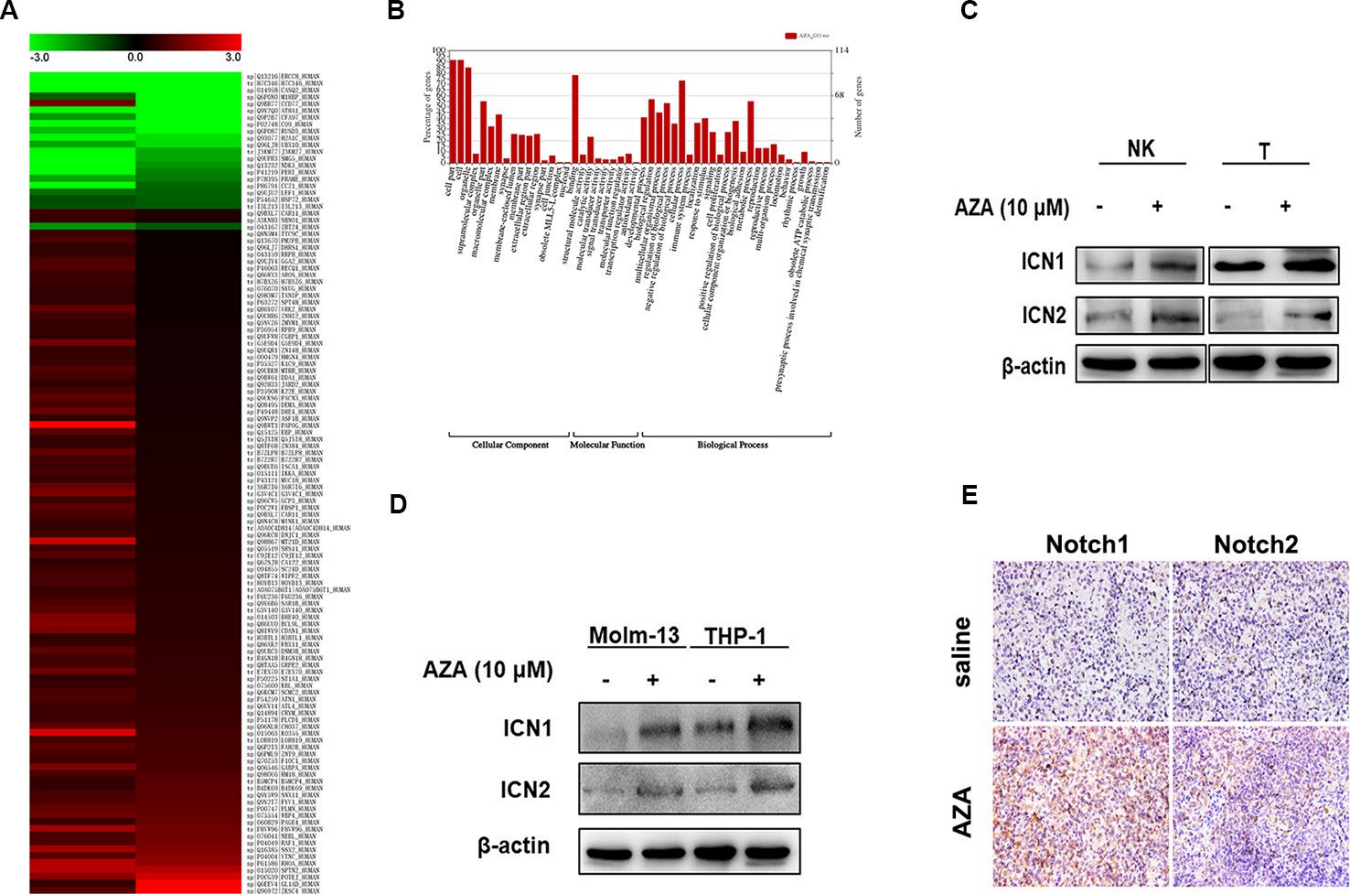
Notch was found up-regulated after azelaic acid (AZA) treatment. **(A)** 120 differential expressed proteins associated in 52 functions after AZA treatment by protein mass spectrometry analysis in the heat map. **(B)** GO annotation with 528 differential expressed proteins. **(C, D)** THP-1, Molm-13, NK, and T cells were treated with 10 µM AZA for 24 h, the expression level of ICN1 and ICN2 was validated by western blot. **(E)** The expression of Notch1 and Notch2 in mouse spleen measured by ICH. IHC, immunohistochemistry.

To clarify the exact mechanism of AZA, we performed Protein Mass Spectrometry Analysis and WEGO Analysis. A total of 528 differential expressed proteins (DEPs) were filtered depending on two independent peptides with 95% confidence. The detailed method has been described previously (Jin et al., 2018). Briefly, total proteins are listed in **Additional File 3**. Importantly, the 528 DEPs were annotated according to the function by WEGO Analysis (**Figure 5B**). One hundred and twenty DEPs involved in DNA damage and DNA repair, cell growth, cytotoxicity, immune response, and antioxidant activity were analyzed by heat map (**Figure 5A**), whilst 10 DEPs were involved in the immune system development, immune effector process, positive and negative regulation of immune system process, leukocyte activation, activation of immune response, and immune response. The detailed information was screened in **Additional File 4**.

Furthermore, we found that the expression levels of the Notch1 and Notch2 proteins were significantly up-regulated. The Notch signaling pathway is an important regulator of immune cell development and function (Radtke et al., 2013). Hence, we analyzed the proteins that interact with Notch1 by STRING v11.0 (**Additional File 5, Figure S2**), and then detected the expression level of Notch1 and Notch2 in AZA-treated AML cell lines and NK and T cells. As shown in Fig5, the intracellular Notch, the activated form of Notch (ICN), was increased after the administration of AZA, as shown by western blotting (**Figure 5C**), the Notch expression in NK and T cells were similarly increased in NK and T cells (**Figure 5D**). Similar results were obtained by immunohistochemistry in the AML model (**Figure 5E**).

### AZA Exerts Antileukemic Effect *via* Activation of Notch Signaling Pathway

**Figure 6:**
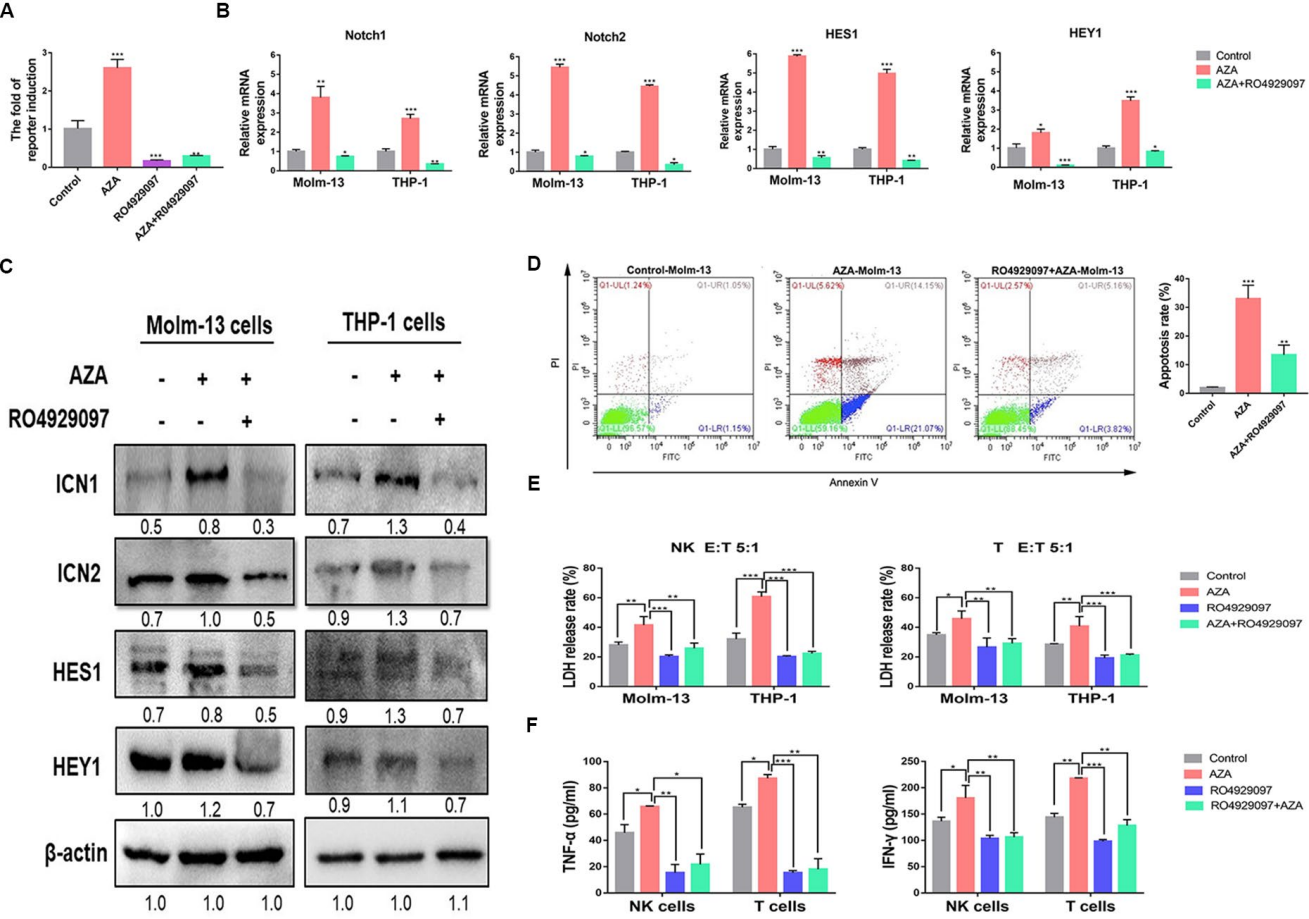
Azelaic acid (AZA) exerts anti-leukemic effect by activating the Notch signaling pathway. **(A)** Notch responsive elements were transfected into 293T cells after 24 h. Cells were then treated with 10 µM AZA, 10 µM RO4929097, and combination for 24 h, the Notch signaling reporter assay was measured by dual luciferase reporter activity. **(B)** Validation of the RNA expression of Notch1 and Notch2, the downstream target genes *HES1* and *HEY1* in Molm-13 and THP-1 cells by qPCR. **(C)** The protein expression level of ICN1, ICN2, HEY1, and HES1 in acute myeloid leukemia (AML) cell lines after treatment of AZA and RO4929097 and their detection by western blot. ImageJ was used for the densitometric analysis. Data represent means ± SD. **(D)** Molm-13 cells were pretreated with 10 µM RO4939097 for 24 h, and then treated with 10 µM AZA for another 24 h. Cells were collected for apoptosis analysis. **(E)** NK cells and T cells were pretreated with 10 µM AZA, 10 µM RO4929097, and combination for 24h before co-culture with THP-1 cell and Molm-13 cells at an E:T ratio of 5:1. The cytotoxicity of NK and T was determined by detecting the LDH release rate. **(F)** NK and T cells were pre-treated with AZA and RO4929097, then co-cultured with THP-1 cell at an E:T ratio of 3:1 for 4 h. The level of TNF-α and IFN-γ in the supernatant was measured by ELISA. A Total of three independent experiments were performed. *P < 0.05, **P < 0.01,***P < 0.001.

To confirm that the Notch signaling pathway was activated by AZA to evaluate the effect of AZA on Notch activation, we detected the activity of the Notch signaling pathway in 293T cells expressing CBF1/RBP-Jκ luciferase reporter vector by luciferase reporter assay. We found that, AZA remarkably increased the activity of Notch signaling pathway compared to the single use of Notch inhibitor, RO4929097, and the combination (**Figure 6A**). We proceeded to perform quantitative real-time PCR to confirm this finding. As a result, AZA was found to be able to induce higher Notch1 and Notch2 expression levels. Moreover, the activation of Notch signaling pathway can induce the upregulation of the Notch downstream target gene *HES1* and *HEY1* (**Figure 6B**). Interestingly, the following western blotting results also confirmed that AZA induced the activation of Notch signaling pathway (**Figure 6C**).

To identify whether AZA-mediated cytotoxicity was caused by Notch activation, we pharmacologically inactivated endogenous Notch with RO4929097 and assessed the eﬀect of AZA on apoptosis. Molm-13 cells were pretreated with RO4929097 for 24 h, then treated with AZA for apoptosis analysis. The results suggested that AML cells were resistant to AZA when preinactivated with Notch (**Figure 6D**). We proceeded to measure the cytotoxicity against AML cells of NK and T cells pre-treated with AZA and RO4929097 and RO4929097 combined with AZA. Following the inhibition of Notch by RO4929097, the cytotoxic activity of NK and T cell was attenuated in RO429097 group; as shown in **Figure 6A**, the strong inhibition caused by RO4929097 cannot be reversed by AZA, and as such, the cytotoxicity of NK and T cells in RO4929097 combined with AZA group was also attenuated (**Figure 6E**). Finally, we analyzed the levels of IFN-γ and TNF-α in the supernatant of NK and T cell after treatment with RO4929097 and AZA. The IFN-γ and TNF-α levels were dramatically decreased in RO4929097 group and RO4929097 combined with AZA group in contrast to the AZA group (**Figure 6F**). The results sufficiently demonstrated that AZA mediated the cytotoxic activity *via* the activation of Notch.

### AZA Inhibits the Tumorigenicity of AML and Prolongs Survival *In Vivo*

**Figure 7:**
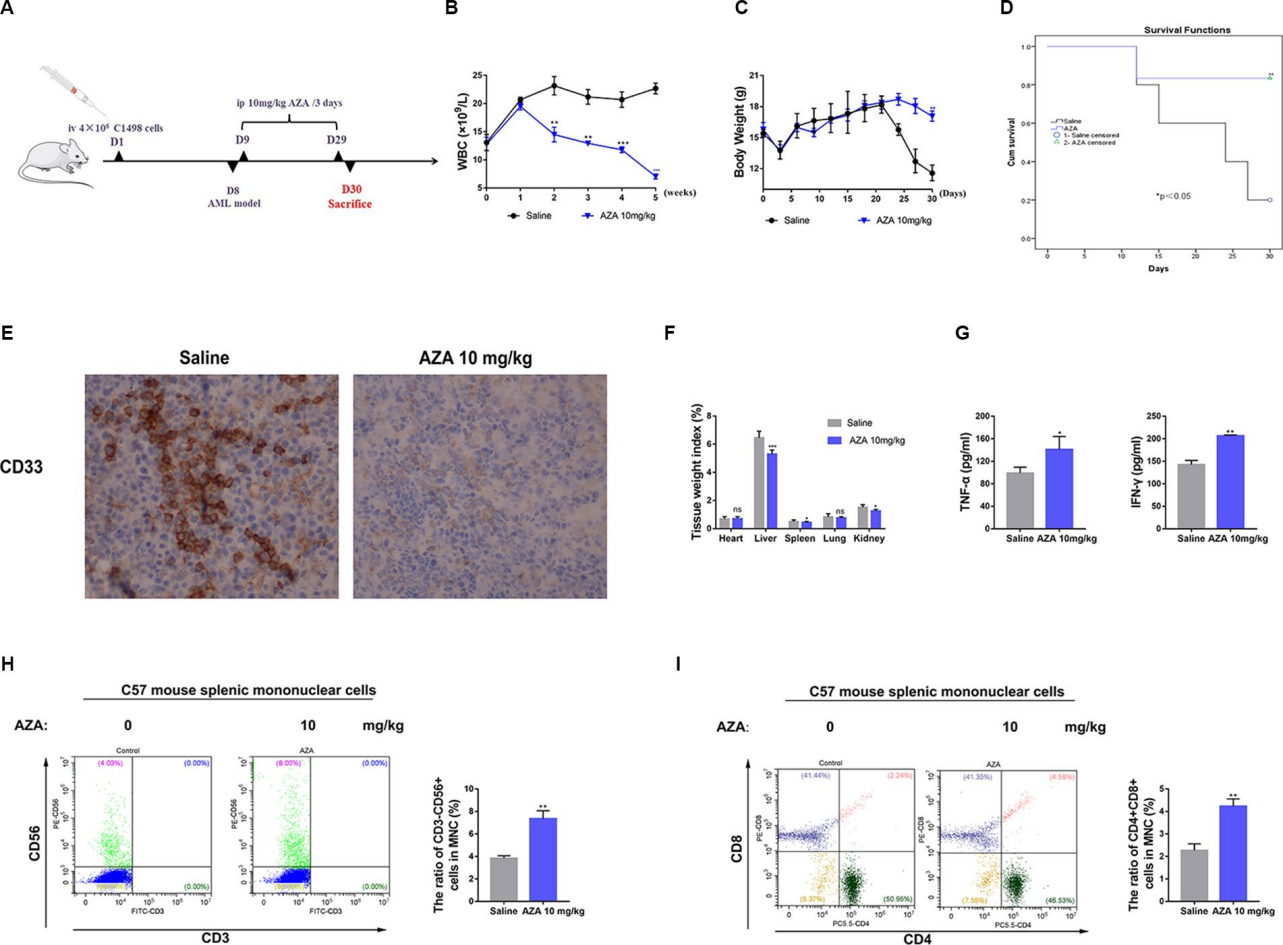
Azelaic acid (AZA) inhibits the tumorigenicity of acute myeloid leukemia (AML) *in vivo*. **(A)** The construction of leukemia-bearing mice model and the therapeutic pattern. **(B)** Body weight change. **(C)** White blood cell counting. **(D)**: Survival analysis. **(E)** Leukemia cells showed spleen infiltration by the ICH method.**(F)** Tissue weight index. **(G)** The level of TNF-α and IFN-γ in plasma. **(H)** Mouse MNCs were stained with CD3 and CD56 for flow cytometric analysis. Right, the percentage of positive staining cells. **(I)** Mouse MNCs were stained with CD3, CD4, and CD8 for flow cytometric analysis. Right, the percentage of positive staining cells. MNCs: the splenic mononuclear cells.

To assess whether AZA can be successfully applied *in vivo* to treat AML, we constructed an AML mouse model using C1498 cell as reported previously (Yan et al., 2018). Briefly, C1498 cells (4×10^6^ per mouse) were injected into 4–5 weeks old C57BL/6 female mice through the tail-vein (n = 12, six mice per group), when the WBC counts indicated the illness, the model was successfully established. AML cells were also observed in peripheral blood smear (**Additional file 6: Figure S3A**). Additionally, AZA were administered every 3 days for three weeks and the administration of saline was used as a negative control. Mice were sacrificed 4 weeks later, and their tissues (heart, liver, spleen, lung, kidney, bone marrow) and blood were harvested for a pathological study (**Figure 7A**). Consistent with its inhibitory effects on AML cells *in vitro*, AZA could remarkably reduce the number of WBCs (**Figure 7C**) and extend the overall survival of leukemia mice compared to the control group (**Figure 7D**). Furthermore, body weight of leukemia mice in the saline group was dramatic decreased (**Figure 7B**) and trichomadesis was observed (**Additional file 6: Figure S3B**) due to the leukemic disease. However, spleen weight index was decreased (**Figure 7F**) and the leukemic spleen infiltration was relieved in the AZA group suggesting the disease remission (**Figure 5E**, **Figure S3C**).

To further test the immunomodulatory effect of AZA *in vivo*, we harvested mouse spleen MNCs and plasma. IFN-γ and TNF-α levels in the plasma secreted in AZA-treated group were significantly higher than those in the saline group (**Figure 7G**). The proportion of CD3^-^CD56^+^NK cells (**Figure 7H**) and CD4^+^CD8^+^T cells (**Figure 7I**) were significantly increased after AZA treatment, but the proportion of CD3^+^CD4^+^T cells and CD3^+^CD8^+^cells did not show a clear change (**Additional file 6: Figure S3D**). Thus, we inferred that AZA can enhance the cytotoxicity of effector T cells *in vivo*, but it had no effect on the differentiation of the T cell subsets. Taken together, these results indicated that AZA represses AML tumorigenicity *in vivo*.

## Discussion

Although targeted drugs and the novel immunotherapies such as chimeric antigen receptor T cells (CAR-T) and immune checkpoint inhibitors (ICI) have achieved meaningful success in the treatment of some hematologic malignancies, due to lack of leukemia-specific cell surface antigens and the lowest mutational burdens, targeted therapy, CAR-T and ICI have only limited effect on AML (Beyar-Katz and Gill, 2018). The overall survival of AML remains very poor. Therefore, identification of novel agents is necessary.

AZA was revealed to have a cytotoxic action on many tumor cells (Schulte et al., 2015), a previous study showed that AZA had antileukemic activity in different types of AML cells, and normal cells were found to be unaffected at the same dose (Pan et al., 2017). The present findings verified the antileukemic effect of AZA and discovered its immunoregulatory effect for the first time. A further mechanistic analysis identified the increase in Notch expression after AZA treatment by quantitative proteomics, qPCR, Western blot, dual luciferase reporter gene assays, and immunohistochemistry. Importantly, these results support the claim that AZA suppresses the proliferation of AML cell and enhances the cytotoxicity of NK and T cells against AML by activating the Notch signaling pathway.

Although studies demonstrated that *Notch* was an oncogene in many tumors (Gu et al., 2012), and Notch1 was exclusively oncogenic in hematological malignancies such as T cell acute lymphoblastic leukemia (Aifantis et al., 2008), recent studies suggest that Notch was a tumor suppressor in AML. The activation of Notch can inhibit leukemogenesis through the downstream target (Klinakis et al., 2011; Kannan et al., 2013). In addition, Notch played a crucial role in regulating the development and cytotoxicity of NK and T cells (Radtke et al., 2013). We also found that Notch promotes the development and late stage maturation of NK cells (DeHart et al., 2005). Additionally, the activation of Notch enhanced the upregulation of CD16 and killer Ig–like receptor (KIR), which is concomitant with the increased cytolytic effector capacity such as the upregulation of CD107a and the secretion of TNF-α and IFN-γ (Kijima et al., 2008; Felices et al., 2014). Notch participated in early T cell development (Radtke et al., 2004), activation, CTL differentiation (Osborne and Minter, 2007), and immune response (Wong et al., 2003). Notch can directly regulate the expression of granzyme B and perforin in CD8^+^T cell (Cho et al., 2009); additionally, Notch also promotes the cytotoxicity of CD8^+^T cell by the transcription factors RBP-J and CREB1(Maekawa et al., 2008). Interestingly, the activation of Notch enhanced the cytolytic activity of CD8^+^T cell by increasing the secretion of TNF-α, IFN-γ, and granzyme B (Kuijk et al., 2013). TNF-α suppressed the proliferation of AML cells and patient myeloid leukemia cells effectively, IFN-γ can act in in synergy with TNF-α (Geissler et al., 1989). The abnormalities of the immune microenvironment in AML contributed to the dysfunction of NK and T cells, which affected the immunosurveillance (Barrett and Le Blanc, 2010), this was one of the main reasons for the relapse and poor prognosis of AML patients (Lamble and Lind, 2018). Thus, reactivation of Notch can enhance the cytotoxicity and the secretion of TNF-α and IFN-γ of NK and T cells against AML.

Identification of the Notch agonist is of great interest. To date, pharmacological Notch activation like lentivirus or peptide-mediated Notch reactivation is still associated with several problems such as low bioavailability and peptide instability (Ye et al., 2016). Importantly, the combination of nontoxic, nonteratogenic, and nonmutagenic properties of AZA encourages its promising Notch agonist function (Breathnach et al., 1989), thereby allowing AZA to inhibit the proliferation of AML cells. Furthermore, from the present study, we found that AZA enhances the sensitivity of chemotherapy (Pan et al., 2017), AZA is expected to be a novel agent for the treatment of AML. However, further research on its effectiveness when used alone or in combination with other agents is encouraged.

This study provides a potential therapeutic approach in AML through activating the Notch signaling pathway, which proves beneficial in giving some insights on the treatment of some cancers where Notch servers as a tumor suppressor (Lobry et al., 2011).

## Conclusion

AZA exerts anti-leukemic effect by activating the Notch signaling pathway. The activation of this pathway does not merely act as a tumor suppressor in AML; but also promotes the proliferation and activation of NK and T cells, which increase the secretion of cytotoxic cytokines against AML. AZA is also a promising agent against AML, and reactivating the Notch signaling pathway may provide a new strategy in the treatment of AML.

## Data Availability Statement

The raw data supporting the conclusions of this manuscript will be made available by the authors, without undue reservation, to any qualified researcher.

## Ethics Statement

The studies involving human participants were reviewed and approved by the Ethics and Scientific Committee of Zhongnan Hospital of Wuhan University. The patients/participants provided their written informed consent to participate in this study. The animal study was reviewed and approved by the Animal Experiment Center of Wuhan University.

## Author Contributions

FZ provided the project direction. DZ performed the experiments, analyzed the data, and wrote the manuscript until the final submission version. YJ guided the experiments. QY and TY contributed to the cell experiments. YC, LX, BW, and ZL contributed to the animal experiments. YL helped synthesizing the BDP-AZA, YL and LLia helped guiding the single-cell microfluidics experiments. AX, LD, HJ, HT, and YT collected the clinical samples from the AML patients. XYL and HS helped analyzing the peripheral blood smears, HE and ICH (hematoxylin and eosin, immunohistochemistry), and LLiu helped performing the flow cytometry and analyzing the results. YJ, YW, and YP revised themanuscript. All authors read and approved the final manuscript.

## Funding

This work was supported by the Natural Science Foundation of China (NSFC) program (grant number 81770179) and the Hong Kong Scholars Program (No. XJ2018060).

## Conflict of Interest

The authors declare that the research was conducted in the absence of any commercial or financial relationships that could be construed as a potential conflict of interest.

## Supplementary Material

Data Sheet 1 The synthesis and characterization of BDP-AZA.

Data Sheet 2 The primer sequence used for RT-PCR.

Data Sheet 3 Differential expressed proteins which were related to immunity identified by MS after AZA treatment.

Figure S1 The excitation wavelength and emission wavelength of BDP-AZA.

Figure S2 The interaction between Notch and other proteins was analyzed by STRING.

Figure S3 **(A)** Analysis of peripheral blood smear after the injection of C1498 cells. **(B)** The mice in saline group displayed trichomadesis in compared to AZA group. **(C)** Analysis of spleen HE staining after AZA treatment. **(D)** Mouse MNCs were stained with CD3, CD4 and CD8 for flow cytometric analysis.
