# Supplementary material for: Antiproliferative and Immunoregulatory Effects of Azelaic Acid Against Acute Myeloid Leukemia via the Activation of Notch Signaling Pathway
Source: Front Pharmacol. 2019 Nov 29;10:1396. doi: 10.3389/fphar.2019.01396 (PMC6901913; doi:10.3389/fphar.2019.01396)
Supplement: Data Sheet 1 — The synthesis and characterization of BDP-AZA. [file DataSheet_1.docx]

**Additional file 1.** The synthesis and characterization of BDP-AZA.

**Scheme 1** Synthesis of BDP-AZA: 1 a. 2,4-dimethylpyrrole, TFA, DDQ, DCM, room temperature, 8 h; b. Et_3_N, BF_3_ OEt_2_, room temperature, 12 h, 25.75%. 2 EDCI, DMAP, THF, room temperature, 31.4%.

Synthesis of 4,4-difluoro-8-(4-hydroxy)phenyl-1,3,5,7-tetramethyl-4-bora-3a,4a-diaza-s-indacene(BDP-OH)

2,4-Dimethylpyrrole (1.647 mL, 16 mmol) and 4-hydroxybenzaldehyde (0.977 g, 8 mmol) were dissolved in dry CH_2_Cl_2_ (250 mL) under argon atmosphere. trifluoroacetic acid (TFA,120 μL) was added to the solution and reaction mixture was stirred at room temperature for 8 h. After complete consumption of the aldehyde (monitored via TLC), a solution of DDQ (1.816 g, 8 mmol) in dry DCM (100 mL) was added, and the stirring was continued for 0.5 h. Then 16 mL dry triethylamine was added to the mixture and after stirring for 15 min, BF_3_OEt_2_ (16 mL) was added dropwise at 0 ℃. The stirring was continued for 12 h and then the reaction mixture was washed with NaHCO_3_ solution several times and extracted with DCM. The organic phase was dried over MgSO_4_. The solvent was evaporated and the residue was purified by column chromatography (silica gel, DCM) to obtain a red solid 0.70 g (25.75%).

Synthesis of 4,4-difluoro-8-(9- phenyl azelate)-1,3,5,7-tetramethyl-4-bora-3a,4a-diaza-s-indacene(BDP-AZA)

AZA (0.094 g, 0.5 mmol) was dissolved in THF (10 mL). Then EDCI (0.095 g, 0.5 mmol ) and DMAP( 0.012 g, 0.1 mmol) were added to the solution. After 12 h, BDP-OH (0.085 g, 0.25 mmol) was added followed by string at room temperature for 4 h. The solvent was removed under reduced pressure. The crude product was puriﬁed with silica-gel column chromatography, 0.04 g red solid powder was obtained, yield: 31.4%.


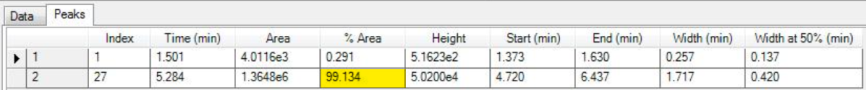

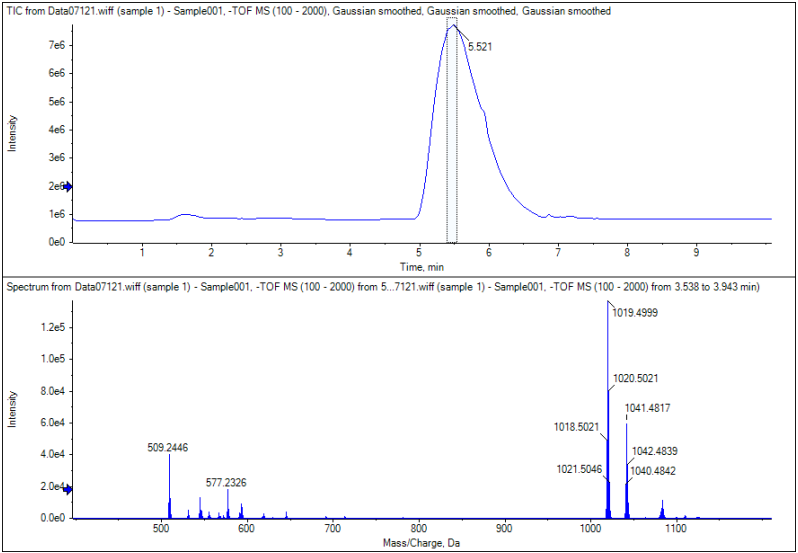


(509 for [M-H]^-^, 1019 for [2M-H]^-^, 1042 for [2M-H+Na]^-^ )
