# Supplementary material for: Antiproliferative and Immunoregulatory Effects of Azelaic Acid Against Acute Myeloid Leukemia via the Activation of Notch Signaling Pathway
Source: Front Pharmacol. 2019 Nov 29;10:1396. doi: 10.3389/fphar.2019.01396 (PMC6901913; doi:10.3389/fphar.2019.01396)
Supplement: Data Sheet 2 — The primer sequence used for RT-PCR. [file DataSheet_2.docx]

**Additional file 2.** The primer sequence used for RT-PCR.

| Gene | Forward | Reverse |
| --- | --- | --- |
| *Notch1* | 5ʹ-AGGCGTGGCAGACTATGC-3ʹ | 5ʹ-TTGTACTCCGTCAGCGTGA-3ʹ |
| *Notch2* | 5ʹ-CAACCGCAATGGAGGCTATG-3ʹ | 5ʹ-GCGAAGGCACAATCATCAATG-3ʹ |
| *HES1* | 5’-ATGGAGAAAAATTCCTCGTCCC-3' | 5'-TTCAGAGCATCCAAAATCAGTGT-3' |
| *HEY1* | 5'-GAAACTTGAGTTCGGCTCTAGG-3' | 5'-  GCTTAGCAGATCCTTGCTCCAT-3' |
| *GAPDH* | 5’-TGATGACATCAAGAAGGT GGTGAA -3’ | 5’-TCCTTGGAGGCCATGTGG GCC AT -3 |
