# Supplementary material for: Antiproliferative and Immunoregulatory Effects of Azelaic Acid Against Acute Myeloid Leukemia via the Activation of Notch Signaling Pathway
Source: Front Pharmacol. 2019 Nov 29;10:1396. doi: 10.3389/fphar.2019.01396 (PMC6901913; doi:10.3389/fphar.2019.01396)
Supplement: Data Sheet 3 — Differential expressed proteins which were related to immunity identified by MS after AZA treatment. [file DataSheet_3.docx]

**Additional file 3.** Differential expressed proteins which were related to immunity identified by MS after AZA treatment.

| **Biological Process** | **Differentially expressed protein** |
| --- | --- |
| immune system development (7) | Picalm,Rpl22, Sart1,Notch2,Notch1,Dmtn,Prrc2c |
| immune response (4) | Notch2, Notch1, Mavs, Hist2h2be |
| immune effector process (4) | Mavs, Notch2, Notch1, Sart1 |
| positive regulation of immune system process (3) | Mavs, Notch2, Sart1 |
| activation of immune response (1) | Mavs |
| leukocyte activation (3) | Notch2, Sart1, Rpl22 |
| myeloid cell homeostasis (1) | Dmtn |
| negative regulation of immune system process (1) | Notch1 |
| immune response (4) | Mavs, Notch2, Notch1, Hist2h2be |
| positive regulation of immune system processes (3) | Mavs, Notch2, Sart1 |
