# Supplementary figures and images for: Antiproliferative and Immunoregulatory Effects of Azelaic Acid Against Acute Myeloid Leukemia via the Activation of Notch Signaling Pathway
Source: Front Pharmacol. 2019 Nov 29;10:1396. doi: 10.3389/fphar.2019.01396 (PMC6901913; doi:10.3389/fphar.2019.01396)

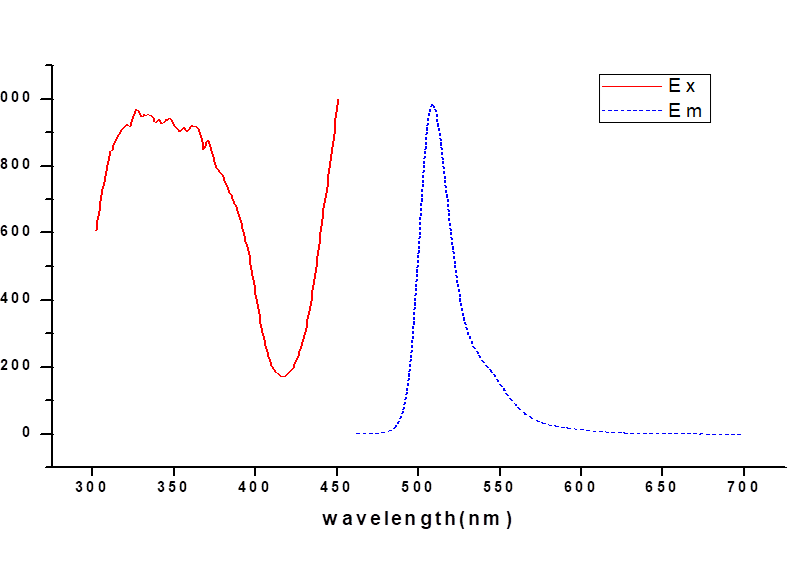

Supplement: Figure S1 — The excitation wavelength and emission wavelength of BDP-AZA. [file Image_1.png]

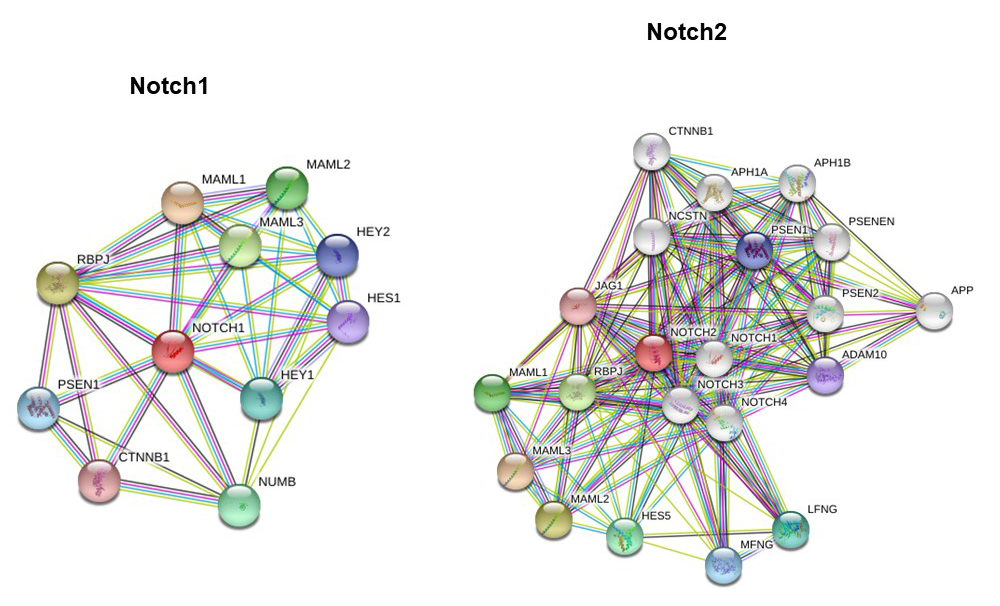

Supplement: Figure S2 — The interaction between Notch and other proteins was analyzed by STRING. [file Image_2.tif]

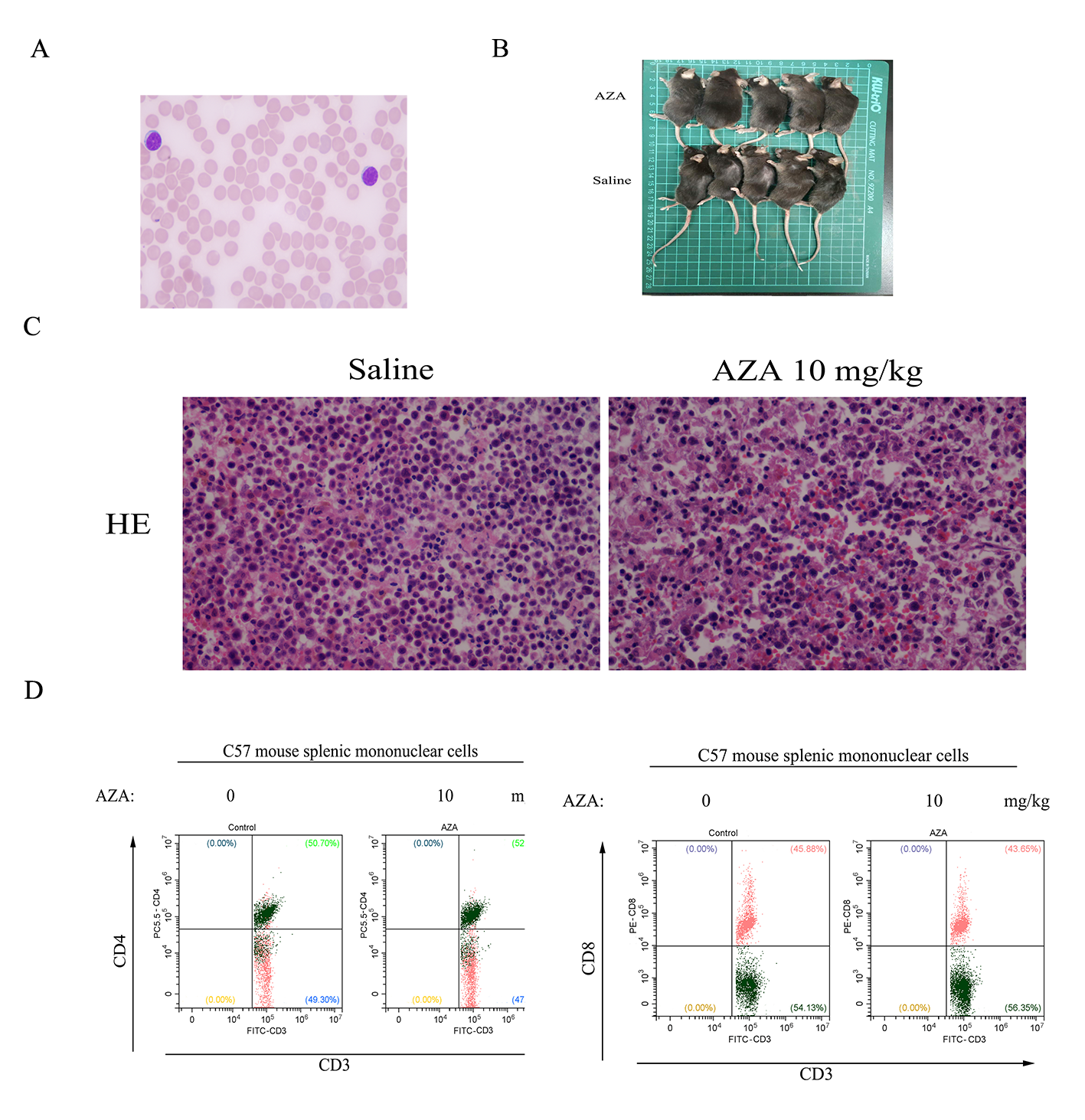

Supplement: Figure S3 — (A) Analysis of peripheral blood smear after the injection of C1498 cells. (B) The mice in saline group displayed trichomadesis in compared to AZA group. (C) Analysis of spleen HE staining after AZA treatment. (D) Mouse MNCs were stained with CD3, CD4 and CD8 for flow cytometric analysis. [file Image_3.tif]
